# Mental Health in the Mediterranean Area

**DOI:** 10.2174/1745017902016010067

**Published:** 2020-07-30

**Authors:** Mauro Giovanni Carta, Mehmet Eskin, Driss Moussaouiand, Elie Karam

**Affiliations:** 1 Department of Medical Sciences and Public Health, University of Cagliari, Cagliari, Italy; 2 Koç Üniversitesi, İnsani Bilimler ve Edebiyat Fakültesi, Psikoloji Bölümü, Sarıyer,İstanbul, Turkey; 3 IbnRushd University, Psychiatric Centre, Casablanca, Morocco; 4 Department of Psychiatry & Clinical Psychology, Institute for Development, Research, Advocacy and Applied Care (IDRAAC), Medical Institute for Neuropsychological Disorders (MIND), St. George Hospital University Medical Center, Faculty of Medicine, University of Balamand, Beirut, Lebanon

## 1 INTRODUCTION: A HISTORICAL PERSPECTIVE

The theme of “Mental Health and Mediterranean” opens the floor to three levels of discussion: 1) the relevance of the area in the current framework; 2) the significance of whole scientific communication between the shores of the Mediterranean in a historical perspective in the current context and, finally, 3) the specific role of the exchanges in mental health and between the actors of mental health care.

Recently, the Mediterranean has become once again one of the critical regions of the world. The area has become a theater of conflicts and is affected by the consequences of the crisis from the surrounding areas. Violence against populations, forced migration, problems in the reception of asylum seekers, ethnic and religious discrimination and conflicts are some of the adverse consequences that imply (not only but also) public health issues [1]. The repercussions on the mental health of communities may have an impact over time to come. Knowledge of nearby cultures and its exchange by scientists are therefore essential.

Once the Mediterranean was the center of civilization, but today it has been reduced to blue beaches, diet and epicenter of human tragedies. Having said this, the region should be something more than this. Considering the fact, scientific communities of the Mediterranean region must regain the will and strength to communicate and to establish links in this period of crisis.

In fact, in contacts and relationships between communities of the shores of the Mediterranean (north-south, east-west) science has always played a role. Scientific exchanges were also often the consequence of wars. But even then, the periods of maximum expansion and well-being of some Mediterranean exchanges were also quite common. Suleiman the Magnificent and his predecessors invited architects and artists of the Italian Renaissance to Istanbul as reported by Vasari and as more recent historical research has confirmed [2, 3]. A single-deck bridge spanning the Golden Horn design was found among Leonardo da Vinci's own documents, and it is now preserved in the National Library of France; it was considered for a long time a simple fruit of the fruitful imagination of the master. More recent historical discoveries have confirmed that there had been a real invitation from Suleiman to Leonardo to submit plans to unify Asia and Europe. Creating a (real) bridge between north and south and between east and west with the contribution of the scientists of the time which were not asked to espouse a creed but simply for the exchange of scientific skills used for the public good is indeed an ancient project, which does not necessarily start from the now western societies.

These exchanges had been even closer in the field of medical sciences and in the treatment of psychiatric diseases in particular. The Mediterranean became the cradle of medical science and psychiatry through a long journey to which all the cultures of the basin have contributed.

The first structure for hospitalization and treatment of what we now call mental illness was built in ancient Egypt. Greeks found a medical approach to mental illness from Hippocrates. Romans inherited this approach and they produced laws and social rules to regulate the relationship with people with psychosocial disabilities by introducing the concept of non-punishment for mental insanity.

During the Middle Ages, the West lost its medical approach to mental illness and today we think that many of the witches burned at the stake in these centuries were possibly people with mental health issues. In fact the Malleus Maleficarum, the text specifically written for the identification of witches describes “evil influences” which today would be interpreted as psychiatric symptoms [4]. In those dark centuries, the east and the south saved the vision of mental suffering as a disease to be cured. Firstly, far from the Mediterranean, in the Persian courts, it flourished (10^th^-13th centuries) in the Islamic countries of the Mediterranean [5]. The doctors of those tolerant courts were not only Muslims but also, Catholic and Jewish. Moises Maimonides, the Jewish personal physician of the vizier of Saladin, treated depression as a psychosomatic illness and suggested that emotions could affect physical health [6]. The doctors who moved from the Islamic courts to the maritime republics (the most tolerant states of Christianity), then favored the revival of medicine in the West which began to grow again starting with the Italian Renaissance.

It is not easy in today's cultural crisis of the Mediterranean region given the competing interests of the actors of the region, the influence of global events and the interests of other actors. We envision that the cultural and scientific exchanges among the countries of the Mediterranean region will strengthen the regional identity and also contribute to the regional and global welfare and science.

### 1.1 The Contents of the Special Issue

We have discussed some very demanding tasks in this special issue. This present limited contribution cannot be considered exhaustive. It is the first step towards a path that we hope will continue and grow.

The special issue begins with Eskin's work [7]. This paper analyzes macro data on suicidal behavior in countries of the region. The average standardized suicide rates in the Mediterranean countries (with a stronger trend in the Arab Muslim countries) are lower than those of outside countries. However, the study fails to explain the reasons for these differences therefore other studies will be needed to explain the protective factors, which could be useful and help us in prevention. The paper by the University of Palermo in Sicily [8] deals with migrants in the Mediterranean area with a focus on the aspect of problems due to the consequences of trauma during migration on female migrants. This is a not sufficiently detailed aspect of the problem of migration today in the region.

The issue of refugee women is still on the focus of the work of Ahmad Sa'adSalehAlsheikh Ali [9]. This paper shows the effectiveness of a counseling program on psychological well-being and post-traumatic stress disorder among a group of displaced women in Jordan due to civil war. The results suggest taking into consideration the importance of cultural mediation interventions when working in this field.

YosraZgreb and coll [10] address the issue of staff perception of respect for human rights of users in mental health facilities in four countries (Italy, Tunisia, Gaza and North Macedonia). The authors put forward the hypothesis regarding the perception of professionals working in the mental health care setting and that the rights of users are respected is a determinant of the good organizational climate of the teams. The analysis of the results offers an insider look at the organizational conditions in mental health systems from countries. The results concerning the multi-centric validation of the tool used in this study are presented by the work of Hursky and co-workers [11].

Another paper compares the beliefs about mental disorders in the population of a Mediterranean nation (Tunisia) to that of Germany [12]. In Tunisia, the public was more likely to adopt psychosocial than biogenetic explanations to reject biological treatments and to recommend religious advice for mental illness. The authors underlined the need for professionals to be sensitive to cultural context.

Another work presents the validation of the Arabic Maghreb version of the Mood Disorders Questionnaire, a screening tool for bipolar disorders, comparing the results with similar works in other languages of the regions [13].

The paper of Ventriglio and co-authors is a narrative review that deals with an under debate issue as the emerging evidence on a possible role of the Mediterranean Diet on general well-being and mental health. The article encourages further researches to verify the potential benefits of the Mediterranean diet and healthy food selection for the protection of disorders, such as major depression and anxiety disorders, and to promote health [14]

The paper on “Postpartum Depression in The Arab Region: A Review” [15]by KhubaibAyoub and co-workers takes stock of current research in a theme of social impact. The work is relevant because it discusses the topic of gender health which has recently seen growing contributions in the Arab world. This work can support the policies and future researches in the field, the postpartum depression in the Arab Region, that has not been sufficiently studied so far.

The final paper presents the results of a pilot project to implement the QualityRights program in Tunisia [16]. The program on human rights in mental health started to be introduced also in countries of the Mediterranean area and this is the first work that presents data repeated over time in the context of the region.

## CONCLUSION

The special issue offers a limited contribution but opens up a set of scenarios relating to the bio-psycho-anthropological specificities of mental health in the Mediterranean region and to future aspects that research could pursue which we think can stimulate researchers in the region to establish contacts and cooperative studies.
